# Deep Learning-based Diagnosis and Localization of Pneumothorax on Portable Supine Chest X-ray in Intensive and Emergency Medicine: A Retrospective Study

**DOI:** 10.1007/s10916-023-02023-1

**Published:** 2023-12-04

**Authors:** Chih-Hung Wang, Tzuching Lin, Guanru Chen, Meng-Rui Lee, Joyce Tay, Cheng-Yi Wu, Meng-Che Wu, Holger R. Roth, Dong Yang, Can Zhao, Weichung Wang, Chien-Hua Huang

**Affiliations:** 1 Department of Emergency Medicine, College of Medicine, National Taiwan University, Taipei, Taiwan; 2 Department of Emergency Medicine, National Taiwan University Hospital, No. 7, Zhongshan S. Rd., Taipei, Zhongzheng Dist. 100 Taiwan; 3 Institute of Applied Mathematical Sciences, National Taiwan University, No. 1, Sec. 4, Roosevelt Rd., Taipei, 106 Taiwan; 4 Department of internal medicine, National Taiwan University Hospital, Taipei, Taiwan; 5 NVIDIA Corporation, Bethesda, USA

**Keywords:** Chest Radiograph, Detection, Localization, Deep Learning, Pneumothorax

## Abstract

**Purpose:**

To develop two deep learning-based systems for diagnosing and localizing pneumothorax on portable supine chest X-rays (SCXRs).

**Methods:**

For this retrospective study, images meeting the following inclusion criteria were included: (1) patient age ≥ 20 years; (2) portable SCXR; (3) imaging obtained in the emergency department or intensive care unit. Included images were temporally split into training (1571 images, between January 2015 and December 2019) and testing (1071 images, between January 2020 to December 2020) datasets. All images were annotated using pixel-level labels. Object detection and image segmentation were adopted to develop separate systems. For the detection-based system, EfficientNet-B2, DneseNet-121, and Inception-v3 were the architecture for the classification model; Deformable DETR, TOOD, and VFNet were the architecture for the localization model. Both classification and localization models of the segmentation-based system shared the UNet architecture.

**Results:**

In diagnosing pneumothorax, performance was excellent for both detection-based (Area under receiver operating characteristics curve [AUC]: 0.940, 95% confidence interval [CI]: 0.907–0.967) and segmentation-based (AUC: 0.979, 95% CI: 0.963–0.991) systems. For images with both predicted and ground-truth pneumothorax, lesion localization was highly accurate (detection-based Dice coefficient: 0.758, 95% CI: 0.707–0.806; segmentation-based Dice coefficient: 0.681, 95% CI: 0.642–0.721). The performance of the two deep learning-based systems declined as pneumothorax size diminished. Nonetheless, both systems were similar or better than human readers in diagnosis or localization performance across all sizes of pneumothorax.

**Conclusions:**

Both deep learning-based systems excelled when tested in a temporally different dataset with differing patient or image characteristics, showing favourable potential for external generalizability.

**Supplementary Information:**

The online version contains supplementary material available at 10.1007/s10916-023-02023-1.

## Introduction

A pneumothorax is an abnormal collection of air in the pleural space between the lung and the chest wall. The annual incidence rate of pneumothorax was approximately 7.3 cases per 100,000 individuals [1]; among hospitalized patients, the incidence of pneumothorax was estimated at 22.7 cases per 100,000 admissions every year [2].

Without prompt recognition and management, pneumothorax may evolve into life-threatening tension pneumothorax. Rapid and correct identification of pneumothorax can minimize the risk associated with tension pneumothorax [3] and thus improve patient outcomes.

Because of its advantage in mobility, the portable supine chest radiograph (SCXR) is one of the most common imaging studies performed in the emergency department (ED) and intensive care unit (ICU) [4, 5]. However, the reported sensitivity of SCXR for detecting pneumothorax varied widely, ranging from 9 to 75% [6], indicating the high rate of misses at initial encounters.

Several factors may explain the heterogeneous sensitivity of SCXR in detecting pneumothorax [7–9]. First, the imaging quality may be reduced because of limitations in the patient’s positioning or body habitus. Second, the distribution of free air in the pleural space is variable and highly dependent on the intrathoracic anatomic structure and relevant pathology in the lung parenchyma and pleural space [10]. The subtle imaging findings of pneumothorax in SCXRs require expertise and cautious inspection to detect its presence.

In the current study, we hypothesized that artificial intelligence-based approaches for interpreting portable SCXRs may facilitate physicians in detecting pneumothorax with greater efficiency and accuracy. We aimed to develop and validate deep learning (DL)-based computer-aided diagnosis (CAD) systems that enable more efficient and accurate pneumothorax detection and localization by portable SCXR.

## Materials and Methods

### Study Design and Setting

We conducted a retrospective study to develop and test our CAD systems in chronologically differing image datasets. Local portable SCXRs were retrieved from the Picture Archiving and Communication System (PACS) database of the National Taiwan University Hospital (NTUH). This study was approved by the Research Ethics Committee of NTUH (reference number: 202003106RINC) and granted a consent waiver. Our results are reported according to the Checklist for Artificial Intelligence in Medical Imaging (CLAIM) [11].

### Image Acquisition and Dataset Designation

**Fig. 1:**
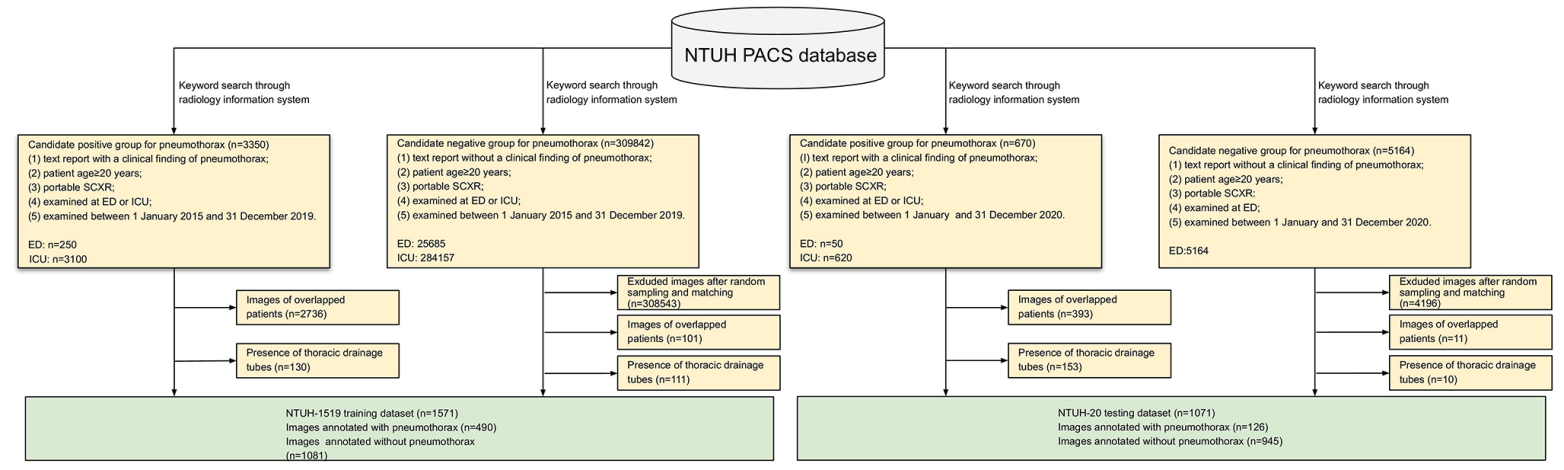
Flow chart of image inclusion process and dataset designation. SCXR, supine chest X-ray; ED, emergency department; ICU, intensive care unit; NTUH, National Taiwan University Hospital; PACS, Picture Archiving and Communication System

As shown in Fig. 1, a Radiology Information System served to identify candidate images used in the building of training (NTUH-1519) and testing (NTUH-20) datasets.

Inclusion criteria for the candidate positive group of NTUH-1519 were as follows: (1) text report with clinical finding of pneumothorax; (2) patient age ≥ 20 years; (3) portable SCXR; (4) imaging obtained in ED or ICU; and (5) exam performed between January 1, 2015 and December 31, 2019. We only selected the first study as the representative image for each patient in the analysis. Inclusion criteria for the candidate negative group were the same as those above, except for text reports devoid of pneumothorax. We randomly selected qualifying images from negative candidates, populating positive and negative groups at an approximate ratio of 1:2. The tentative candidate list was then further scrutinized to avoid overlap of patients.

Image acquisition for NTUH-20 differed in the time frame (January 1, 2020 to December 31, 2020) but was otherwise the same. In addition, the candidate negative group included only images obtained in EDs, and the image ratio for positive and negative groups was approximately 1:10.

We exported all eligible de-identified images in Digital Imaging and Communications in Medicine (DICOM) format, including corresponding text reports for analysis. The radiological reports were generated by various board-certified radiologists for clinical purposes.

### Image Annotation, Ground Truth, and CXR Report Extraction

Each image was first split into 10 × 10 grids of equal size. Bounding boxes were then used to cover the pneumothorax visible in each grid, utilizing the least area. Each image was randomly assigned to two emergency physicians, blinded to each other’s efforts, for image annotation. A total of six board-certified and four board-eligible emergency physicians were involved, each with at least 4 years of clinical experience. All images were ultimately reviewed by an experienced (10 years) board-certified pulmonologist and intensivist who adjusted annotations as necessary. The reviewed annotations served as ground truth in model training and testing. Any images harbouring thoracic drainage tubes were picked up and excluded from further analysis. CXR findings and diagnoses [12, 13] were extracted manually by research assistants blinded to annotations according to the radiology reports.

#### Development of Algorithm

**Fig. 2:**
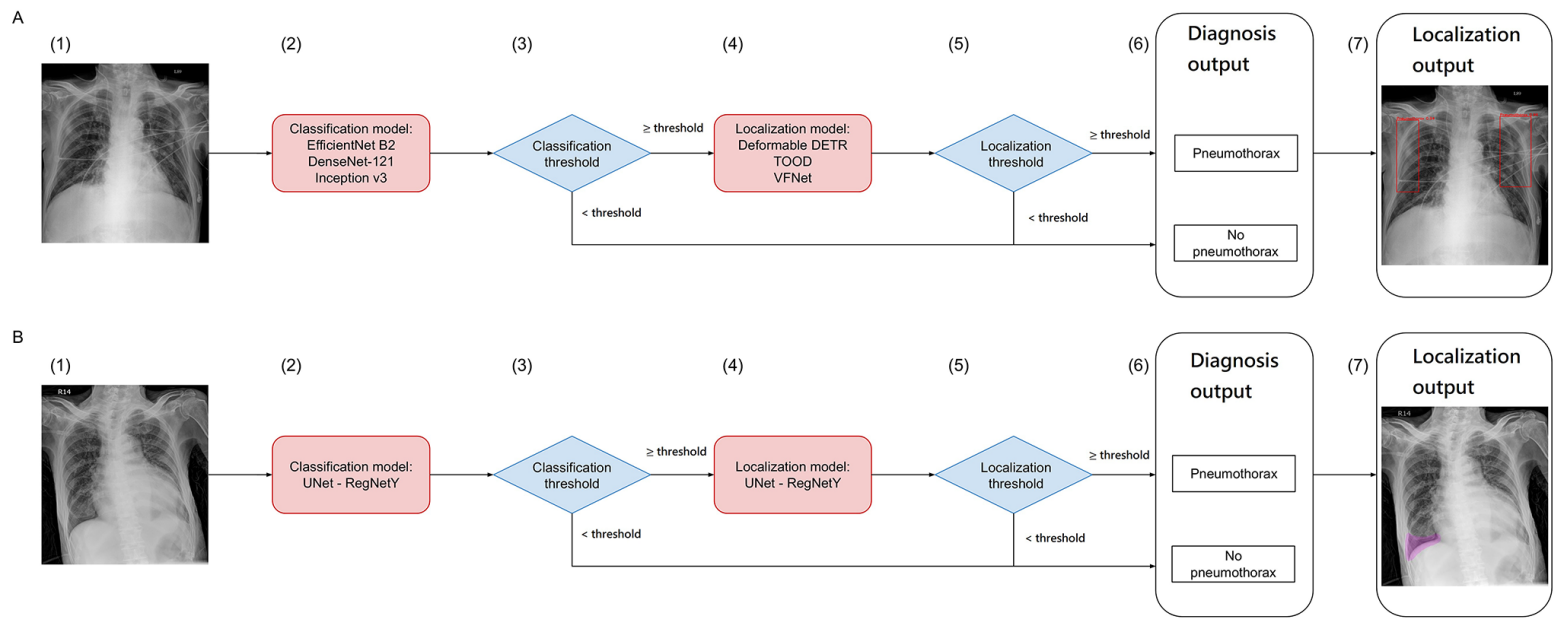
Structures of (**A**) detection-based and (**B**) segmentation-based CAD systems: Each system incorporates a classification model and a localization model, the major difference being the localization method (object detection vs. image segmentation). A CXR image (1) is first passed to the classification model (2) to derive the probability regarding presence of pneumothorax. If the output probability exceeds the classification threshold (3), the image is passed to the localization model (4) to assess pneumothorax position. Once the largest predicted confidence score of the bounding boxes (detection-based CAD system) or predicted areas (segmentation-based CAD system) of the pneumothorax is above the localization threshold (5), the CAD system yields diagnosis (6) and localization (7) outputs. Red rectangles and purple areas denote pneumothorax locations. CAD, computer-aided diagnosis; CXR, chest X-ray

We designed two separate CAD systems (Fig. 2), each including a classification model and a localization model and jointly yielding the following variables: (1) diagnosis output, indicating the presence or absence of pneumothorax, and (2) localization output, indicating the pneumothorax lesion site. Our CAD systems were designated as detection- or segmentation-based systems according to the localization method applied (i.e., object detection or image segmentation).

Supplemental Figs. 1 and 2 show the training pipelines in detail. In brief, NTUH-1519 was first split into different subsets to train the CAD systems. This partition process ensured similar proportions of images (with vs. without pneumothoraces) and eliminated patient overlap across all subsets. All images underwent preprocessing before analysis. The annotated bounding boxes were transformed into segmentation masks for the segmentation-based system. The segmentation masks would also be replaced with one or several larger bounding boxes, covering all adjoining masks at minimal area and serving as input for the detection-based system.

For the detection-based system, EfficientNet-B2 [14], DneseNet-121 [15], and Inception-v3 [16] were selected as the architecture for the classification model; Deformable DETR [17], TOOD [18], and VFNet [19] were selected as the architecture for the localization model. Both classification and localization models of the segmentation-based system shared the UNet [20] architecture, using RegNetY [21] as encoder.

#### Evaluation Metrics of Algorithm

Diagnosis output performance was determined by the area under receiver operating characteristics curve (AUC), area under precision-recall curve, sensitivity, specificity, positive predictive value, and negative predictive value. Youden’s index [22] acquired from the training (NTUH-1519) dataset indicated the optimal threshold for testing.

Localization output performance was measured by Dice coefficient, calculated as twice the area of overlap divided by total pixel count in predicted and ground-truth masks. Dice coefficients were only computed in images positive for both predicted and ground-truth pneumothoraces (i.e., images classified as true positives), referred to as prediction-ground truth TP-Dice. TP-Dice coefficients were also calculated to evaluate the consistency shown by two annotators (inter-annotator TP-Dice).

### Statistical Analysis

Continuous variables were expressed as means with standard deviations while categorical variables as counts and proportions. Continuous variables were compared with Student’s *t*-test, and categorical variables were compared with the Chi-squared test. All statistics were determined as point estimates, with 95% confidence intervals (CIs), through a bootstrap technique at 1,000 repetitions. Prediction-ground truth and inter-annotator TP-Dice coefficients were compared by paired *t*-test. Subgroup analysis was performed to explore the influence of the pneumothorax size on the model performance. The pneumothorax was categorized into large, medium, and small sizes based on the 33rd and 66th percentiles of areas of segmentation masks. A two-tailed *p*-value < 0.05 was considered statistically significant. All computations were driven by open-source freeware (SciPy v1.8.1) [23].

## Results

As shown in Figs. 1 and 2642 images were acquired from the PACS database, (training, 1571; testing, 1071). Significant differences between NTUH-1519 and NTUH-20 datasets are shown in Table 1, with 490 (31.2%) and 126 (11.8%) patients, respectively annotated as pneumothorax. Aside from pneumothorax, other patient characteristics and image findings were numerically similar for images annotated for presence or absence of pneumothorax in NTUH-1519 and NTUH-20 datasets (Supplemental Tables 3 and 4).

**Table 1:** Comparison of training (NTUH-1519) and testing (NTUH-20) datasets

Variables	NTUH-1519 (n = 1571)	NTUH-20 (n = 1071)	*p*-value
Age, year	65.1 (16.1)	58.6 (19.9)	< 0.001
Male, n	946 (60.2)	580 (54.2)	0.002
Qualitative findings in radiology reports, n			
Atelectasis	38 (2.4)	26 (2.4)	0.99
Cardiomegaly	656 (41.8)	286 (26.7)	< 0.001
Consolidation	331 (21.1)	77 (7.2)	< 0.001
Emphysema	6 (0.4)	4 (0.4)	0.97
Endotracheal intubation	558 (35.5)	135 (12.6)	< 0.001
Haziness	192 (12.2)	72 (6.7)	< 0.001
Infiltration	255 (16.2)	204 (19.0)	0.06
Nodularity	55 (3.5)	46 (4.3)	0.30
Opacification	587 (37.4)	379 (35.4)	0.30
Pleural effusion	418 (26.6)	200 (18.7)	< 0.001
Pneumothorax	484 (30.8)	124 (11.6)	< 0.001
Annotation, n			
Pneumothorax	490 (31.2)	126 (11.8)	< 0.001
Large pneumothorax	164 (10.4)	44 (4.1)	< 0.001
Medium pneumothorax	162 (10.3)	35 (3.3)	< 0.001
Small pneumothorax	164 (10.4)	47 (4.4)	< 0.001
Manufacturer, n			< 0.001
Agfa	1405 (89.4)	113 (10.6)	
Canon	128 (8.1)	30 (2.8)	
Carestream	12 (0.8)	0 (0)	
Philips	5 (0.3)	0 (0)	
Samsung	21 (1.3)	928 (86.6)	
Image pixels	8055937.4 (2898803.0)	7538921.0 (2337194.0)	< 0.001

Data expressed as mean (standard deviation) values or as counts (proportions)

**Fig. 3:**
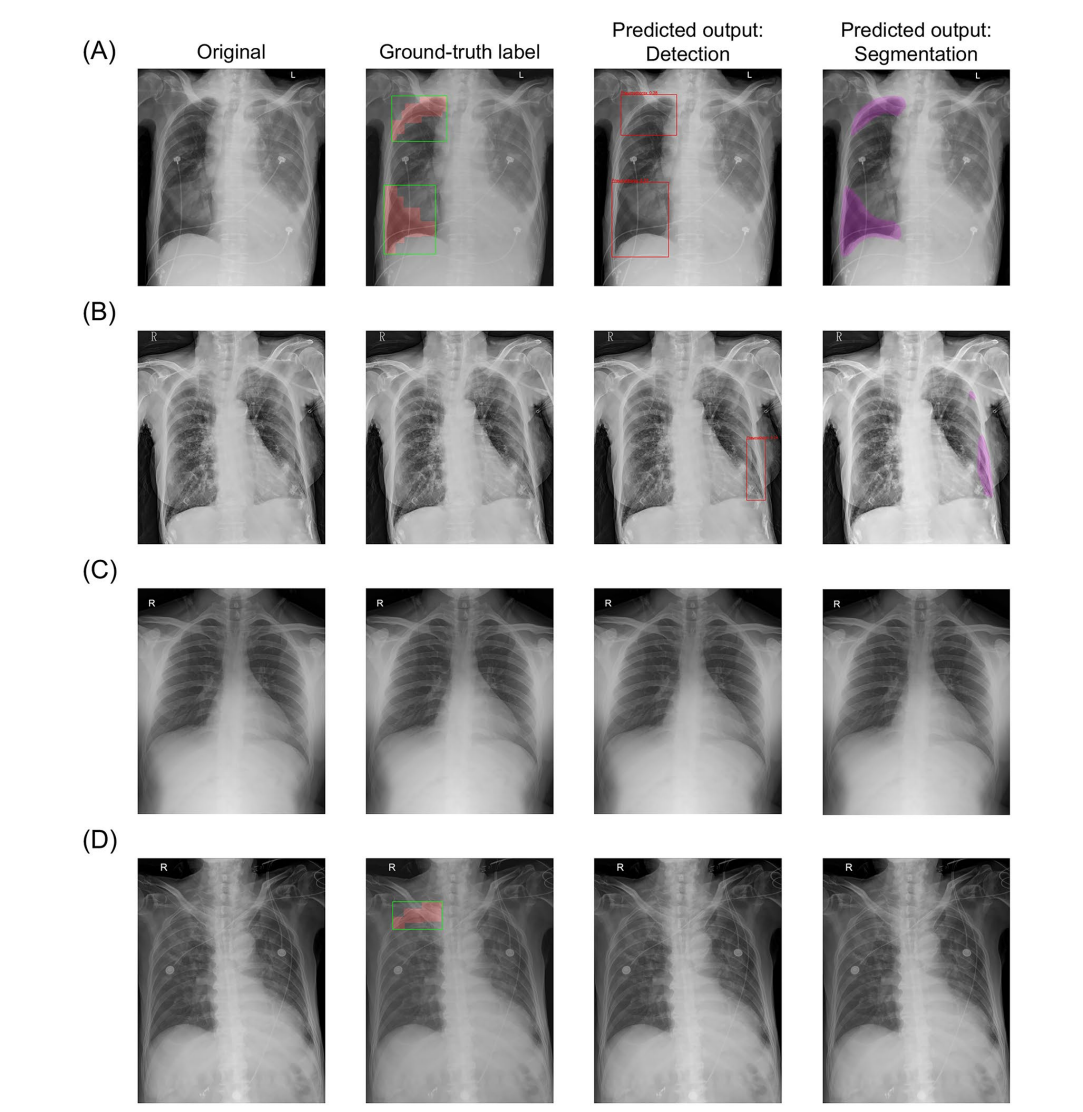
Sample images stratified by predicted results of diagnosis outputs, including (**A**) true-positive, (**B**) false-positive, (**C**) true-negative, and (**D**) false-negative results. The first column at left displays original images. In the second column from left, preprocessed bounding boxes (green rectangles) and segmentation masks (red areas) of detection- and segmentation-based CAD systems are shown. The third column from left demonstrates bounding boxes (red rectangles) predicted by detection-based CAD system, with segmentation masks (purple areas) predicted by segmentation-based CAD system appearing in the fourth column. CAD, computer-aided diagnosis

Table 2 indicates that pneumothorax was accurately diagnosed by detection-based (AUC: 0.940, 95% CI: 0.907–0.967) and segmentation-based (AUC: 0.979, 95% CI: 0.963–0.991) systems, both achieving levels similar to those of radiology reports. Figure 3 demonstrates four representative imaging sets. The overlain predicted bounding boxes or segmentation masks served to assist clinicians in verifying the diagnosis and position of pneumothorax. As shown in Table 3, prediction-ground truth TP-Dice coefficients for detection- and segmentation-based systems were 0.758 (95% CI: 0.707–0.806) and 0.681 (95% CI: 0.642–0.721), respectively, both values significantly surpassing inter-annotator TP-Dice values. Supplemental Table 5 lists the required computational resources for both systems.

**Table 2:** Diagnostic performances of computer-aided diagnosis (CAD) systems and radiology reports

Readers	AUC	AUPRC	Sensitivity	Specificity	PPV	NPV
*Diagnosing pneumothorax*						
Detection-based CAD system	0.940 (0.907–0.967)	0.833 (0.775–0.884)	0.687 (0.598–0.776)	0.991 (0.984–0.996)	0.892 (0.823–0.954)	0.966 (0.955–0.977)
Segmentation-based CAD system	0.979 (0.963–0.991)	0.910 (0.863–0.948)	0.926 (0.874–0.972)	0.926 (0.909–0.942)	0.584 (0.510–0.657)	0.991 (0.985–0.997)
Radiology reports	0.953 (0.927–0.976)	0.866 (0.802–0.923)	0.914 (0.862–0.961)	0.992 (0.986–0.997)	0.936 (0.889–0.976)	0.989 (0.981–0.995)
*Diagnosing large pneumothorax*						
Detection-based CAD system	0.997 (0.993-1.000)	0.950 (0.895–0.987)	0.946 (0.861-1.000)	0.991 (0.984–0.996)	0.788 (0.659–0.903)	0.998 (0.995-1.000)
Segmentation-based CAD system	0.999 (0.998-1.000)	0.977 (0.933-1.000)	1.000 (1.000–1.000)	0.926 (0.909–0.942)	0.336 (0.248–0.426)	1.000 (1.000–1.000)
Radiology reports	0.996 (0.993–0.998)	0.820 (0.697–0.927)	1.000 (1.000–1.000)	0.992 (0.985–0.997)	0.820 (0.697–0.927)	1.000 (1.000–1.000)
*Diagnosing medium pneumothorax*						
Detection-based CAD system	0.947 (0.880–0.991)	0.779 (0.630–0.907)	0.763 (0.600-0.917)	0.991 (0.984–0.996)	0.718 (0.556–0.864)	0.993 (0.987–0.998)
Segmentation-based CAD system	0.976 (0.925–0.999)	0.897 (0.792–0.973)	0.967 (0.889-1.000)	0.926 (0.909–0.942)	0.290 (0.203–0.381)	0.999 (0.997-1.000)
Radiology reports	0.996 (0.993–0.998)	0.791 (0.657–0.923)	1.000 (1.000–1.000)	0.992 (0.985–0.997)	0.791 (0.657–0.923)	1.000 (1.000–1.000)
*Diagnosing small pneumothorax*						
Detection-based CAD system	0.886 (0.822–0.943)	0.478 (0.326–0.637)	0.407 (0.257–0.565)	0.991 (0.985–0.996)	0.652 (0.462–0.833)	0.975 (0.965–0.985)
Segmentation-based CAD system	0.964 (0.942–0.981)	0.636 (0.476–0.778)	0.833 (0.711–0.941)	0.926 (0.909–0.942)	0.330 (0.242–0.420)	0.992 (0.986–0.998)
Radiology reports	0.984 (0.955–0.998)	0.818 (0.698–0.911)	0.975 (0.917-1.000)	0.992 (0.986–0.996)	0.837 (0.723–0.923)	0.999 (0.997-1.000)

Data expressed as point estimates (95% confidence intervals)

AUC, area under receiver operating characteristics curve; AUPRC, area under precision-recall curve; NPV, negative predictive value; PPV, positive predictive value

**Table 3:** Localization performances of computer-aided diagnosis (CAD) systems and annotators

Annotations	Detection-based CAD system			Segmentation-based CAD system		
	Prediction-ground truth TP-Dice	Inter-annotator TP-Dice	*p-*value	Prediction-ground truth TP-Dice	Inter-annotator TP-Dice	*p-*value
Pneumothorax	0.758 (0.707–0.806)	0.717 (0.665–0.766)	< 0.001	0.681 (0.642–0.721)	0.669 (0.653–0.654)	< 0.001
Large pneumothorax	0.883 (0.840–0.921)	0.767 (0.690–0.836)	< 0.001	0.769 (0.715–0.823)	0.756 (0.739–0.741)	< 0.001
Medium pneumothorax	0.708 (0.625–0.798)	0.677 (0.574–0.771)	< 0.001	0.693 (0.628–0.757)	0.639 (0.651–0.653)	< 0.001
Small pneumothorax	0.574 (0.446–0.701)	0.686 (0.596–0.771)	> 0.99	0.558 (0.489–0.627)	0.616 (0.589–0.591)	> 0.99

Data expressed as point estimates (95% confidence intervals)

Prediction-ground truth TP-Dice, coefficient indicating agreement between predicted and ground-truth results; inter-annotator TP-Dice, coefficient indicating agreement between two annotators

Subgroup analysis showed that in diagnosing pneumothorax, performances of both systems declined according to the size (large to small) of pneumothorax, consistent with the trend for radiology reports (Table 2). In terms of pneumothorax localization, diminishing pneumothorax size (large to small) corresponded with similar declines in prediction-ground truth TP-Dice coefficients for both systems, again aligned with the observed trend for inter-annotator TP-Dice values (Table 3).

## Discussion

### Main Findings

Both detection- and segmentation-based systems achieved excellent performance, which was comparable to radiology reports or human annotators. Like human readers, the diagnosis and localization performance of the CAD systems might be influenced by the size of the pneumothorax.

### Annotation of Pneumothorax on SCXR

Most public datasets [24, 25] rely on chest X-rays with image-level labels of common thoracic diseases that are text-mined from radiology reports and are inherently inaccurate [26, 27]. For example, for ChestX-ray14, a study suggested the agreement regarding pneumothorax diagnosis between the image-level label and radiologist review was only about 60% [28], which may lead to poor model generalizability [29].

On the other hand, pixel-based annotation may effectively facilitate the development of pneumothorax-detecting algorithms [30]. For standing CXR, the pneumothorax lesion could usually be delineated [31, 32] by the visceral pleural line in the apicolateral space [33]. Nonetheless, when patients are in the supine position, the spaces where the air is trapped differ from those in the standing position [34]. Adopting segmentation masks to delineate the pneumothorax lesion on SCXR might raise a concern that only those images with clear pleural lines were annotated, leading to selection bias.

Consequently, we used bounding boxes for annotation, allowing for localization of pneumothoraces without distinct pleural lines. Nevertheless, in some lesions, such as those spanning lung apices and basal aspects, the use of bounding boxes might encompass nearly an entire unilateral lung region. This problem was overcome by dividing images into 10 × 10 grids, permitting bounding boxes to accommodate lesions of varying shapes.

### Dataset Selection for Training and Testing Models

Considering the low (0.5-3%) incidence of pneumothorax cited in epidemiologic data [35, 36], use of a consecutive random SCXR sampling for model development may result in class imbalance. Such imbalance may bias CAD systems towards learning features of a more common class (i.e., pneumothorax-negative images) and distort various evaluation metrics [37]. Thus, we employed a case-controlled design [38, 39] to achieve greater balance in training and testing datasets. As shown in Table 1, the higher proportion (31.2%) of images annotated as pneumothorax in the NTUH-1519 dataset may enable our CAD systems to better learn pneumothorax-related features; whereas the lower proportion (11.8%) in NTUH-20 fostered performance testing on a plane approaching real-world prevalence [35, 36].

In a previous study [29], the accuracy of DL-based pneumothorax detection was shown to significantly decline when testing the algorithm in an external dataset. Concerns over accuracy overestimation and limited generalizability of such algorithms may be mitigated by model evaluation in an independent dataset. However, no datasets dedicated to portable SCXRs were available for our purposes. According to the Transparent reporting of a multivariable prediction model for individual prognosis or diagnosis (TRIPOD) statement [40], external validation may use data collected by the same researchers, using the same predictors and outcome definitions and assessments, but typically sampled from a later period (temporal or narrow validation). In our study, the NTUH-20 dataset consisted of SCXRs taken during 2020 at NTUH. Compared with NTUH-1519, NTUH-20 was a chronologically different dataset (2015–2019 vs. 2020), with significant differences (Table 1). According to the TRIPOD statement, the chronologically different testing dataset can be used to verify the external generalizability of the CAD system.

### Diagnosis Output Performance

Niehues et al. [41] used portable SCXR to develop a CAD algorithm with excellent performance in identifying pneumothorax (AUC: 0.92, 95% CI: 0.89–0.95). Nonetheless, the thoracic drains were concomitantly present in approximately half of the images with pneumothorax [41]. It is thus conceivable that these drains were misconstrued in the algorithm as a feature of pneumothorax [42]. Rueckel et al. [30] also collected 3062 SCXRs, including 760 images with pixel-level annotations of pneumothorax and thoracic drain. This model also performed well overall (AUC: 0.877) for unilateral pneumothorax detection.

For the present study, however, we excluded images with thoracic drains and used bounding boxes for pixel-level annotation. Both of detection- and segmentation-based systems delivered excellent performances (AUC values > 0.94) in pneumothorax detection. In our study, the architectures of the classification models differed between the two CAD systems as the UNet-based model [20] itself could output both classification results and localization information.

Routine portable SCXR exams are common practice in critical care [43, 44]. Such regular use of portable SCXR exams may partly account for the prolonged turnaround time from image acquisition to interpretation by a radiologist [45]. Our systems may help prioritize portable SCXRs within queues, flagging those to be checked upfront by a radiologist or earmarking treating clinicians for notifications. As shown in Table 1, there was a high percentage of patients receiving tracheal intubation. Early detection of pneumothorax may facilitate prompt life-saving procedures for these patients to prevent serious complications, such as tension pneumothorax.

### Localization Output Performance

Using standing chest X-rays, a model devised by Lee et al. [46] has achieved a Dice coefficient of 0.798 in pneumothorax localization. Feng et al. [47] also derived a model able to localize the pneumothorax lesion (Dice coefficient: 0.69). Nevertheless, even though Feng et al. [47] included portable SCXRs in the analysis, the researchers excluded films with only supine signs of pneumothorax, e.g., deep sulcus sign. Another model by Zhou et al. [48], based on frontal chest X-rays alone (no portable SCXRs), could detect pneumothorax with a Dice coefficient of 0.827.

The images of portable SCXR are generally deemed suboptimal for diagnosis. The patients are often unable to cooperate during image acquisition, leading to poor bodily orientation or inspiratory efforts. Compared with standing chest X-rays, they are also inferior in image quality, hindering the diagnosis of pneumothorax due to lesser degrees of resolution and luminance [49]. Furthermore, classic findings of pneumothorax on standing chest X-rays are often lacking on portable SCXRs. Given the more challenging interpretation of SCXRs, past models [46–48; 50] may not be suitable for pneumothorax localization on these images.

Both CAD systems we developed (based on object detection or image segmentation) performed excellently in pneumothorax localization, comparable to the level of annotators (Table 3). To the best of our knowledge, our CAD systems may be the first ones capable of localizing pneumothoraces on portable SCXRs. Although the detection- and segmentation-based systems performed similarly in testing, their required computational resources differed substantially (Supplemental Table 5). The detection-based system only outputs approximate positional information with several coordinates of bounding boxes. Logically, its computational demands should be less than those of the segmentation-based system, which provides accurate pixel-wise lesion information. However, the detection-based system must integrate several models for ensemble and thus is more demanding of resources by comparison. Users must take into account specific computational requirements when choosing a preference.

### Influence of Pneumothorax Size

Previous studies [42, 51, 52] have demonstrated that model performance (as with human readings) may be influenced by extent of pneumothorax. A model that Taylor et al. [52] devised correctly identified 100% of large pneumothoraces but only 39% of small ones. Similarly, performance levels of our CAD systems declined as pneumothorax size diminished. This is not surprising, because inter-annotator TP- Dice coefficients also fell as pneumothorax size decreased, underscoring the problematic model learning of small-volume lesions. This phenomenon was more obvious for the detection-based CAD system as its lower prediction-ground truth TP-DICE than the inter-annotator TP-DICE (Table 3) may lead to the lower diagnostic performance for small pneumothorax than the radiology reports (Table 2).

Unlike large pneumothoraces, small pneumothorax is apt to be overlooked by clinicians, especially on portable SCXRs, necessitating assistance by CAD systems. Because most patients subjected to portable SCXRs are those susceptible to complications caused by pneumothorax, especially those receiving mechanical ventilation, timely detection is critical to prevent a small pneumothorax from progressing into tension pneumothorax [53].

### Future Applications

The CAD system can serve two primary functions: (1) prioritizing the SCXRs and selecting those in question to be checked first by the radiologist or (2) issuing notifications to attending clinicians. When the clinicians examine the diagnosis results, the localization outputs of pneumothorax may pop up to facilitate verification of the results. We present the requirements of computational resources for these two CAD systems (Supplemental Table 5), which can assist healthcare institutions in selecting the most suitable model for deployment. Moreover, in future studies, it is warranted to examine the feasibility of adapting these CAD systems for edge computing and their integration into portable chest X-ray machines, which holds the potential to broaden the CAD systems’ applicability.

### Study Limitations

First, because we only have de-identified images available for analysis, we did not know whether patients’ clinical comorbidities may influence the performance of the CAD system. Nonetheless, Table 1 shows there were diverse findings or diagnoses on SCXRs, which might somewhat mitigate this concern. Second, given the low prevalence for pneumothorax [35, 36], we used a case-controlled study design for image collection to ensure sufficient numbers of pneumothorax-positive patients. This design may result in an artificially elevated pneumothorax prevalence in our datasets, compared with real-world settings. We therefore relied on radiology reports or annotators as reader reference points by which to judge CAD system performance. Further prospective studies are warranted to better test performance with real-life pneumothorax prevalence by enrolling consecutive patients from EDs or ICUs on a manageable scale [54].

## Conclusions

We developed two DL-based CAD systems to diagnose and localize pneumothoraces on portable SCXRs, using detection and segmentation methods, respectively. Performances of both systems proved excellent, comparable to those of radiologists or human annotators when tested in a dataset of differing time frame, with differing patient or image characteristics. Hence, the potential for external generalizability seems favourable. Although each performed similar in testing, the detection-based system may demand more in terms of computational resources.

## Electronic Supplementary Material

Below is the link to the electronic supplementary material.

Supplemental Figure 1: Training pipeline for detection-based CAD system: (**A**) During system development, the training dataset (NTUH-1519) was first randomly split into training (80% of training dataset) and holdout (20% of training dataset) subsets. The training subset was then randomly divided into ten equal-sized validation folds (each 8% of training dataset). This partition process ensured that image ratios (with vs. without pneumothorax) were similar among all validation folds and in the holdout subset. The ten validation folds and the holdout subset were used to identify optimal hyperparameters. (**B**) Image preprocessing followed dataset partitioning, modifying image intensity to the photometric interpretation of Monochrome2. CLAHE [55] was applied to increase image contrast. Preprocessed images were subsequently passed to classification and localization models for respective training. (**C**) For the localization model, annotated bounding boxes required further preprocessing, directly transforming the bounding boxes into segmentation masks. The masks were then replaced by one or several larger bounding boxes, covering all adjoining masks within minimum areas for use in training. For classification model, the EfficientNet-B2 [16], DneseNet-121 [15], and Inception-v3 [16] were selected as the model architecture; for localization model, Deformable DETR [17] (backbone: ResNet-50), TOOD [18] (backbone: ResNet-101), and VFNet [19](backbone: ResNet-50) were adopted as the model architecture. The selection of the localization model was based on the comparisons between the state-of-the-art detectors, which were pre-trained on the COCO dataset and fine-tuned on NTUH-1519. We employed commonly used COCO metrics, modifying them to suit the context of image resolution for the assessment. Ultimately, we selected two detectors with the best performance in detecting pneumothorax overall (TOOD and VFNet) and one detector with the best performance in detecting small-sized pneumothorax (Deformable DETR). Supplemental Table 1 presents a comprehensive comparison of the performance of the detectors on NTUH-1519. During the training process, the classification model was trained using Adam at an initial learning rate of 3e^− 4^, a weight decay of 5e^− 4^, and a batch size of 32. For the localization model, Deformable DETR was trained using AdamW and a 60e schedule, whereas TOOD and VFNet were trained using SGD and 2x schedule. A learning rate of 1e^− 4^ was linearly adapted, with a batch size of 16. (**D** & **E**) These loss functions served to supervise the learning process. Diagnosis output of the detection-based CAD system was generated by averaging predicted results of EfficientNet-B2, DneseNet-121, and Inception-v3. Finally, WBF [56] was employed to ensemble predicted results of Deformable DETR, TOOD, and VFNet and produce a localization output. CAD, computer-aided diagnosis; CLAHE, contrast-limited adaptive histogram equalization

Supplemental Figure 2: Training pipeline for segmentation-based CAD system: (**A**) During system development, the training dataset (NTUH-1519) was first randomly split into five equal-sized training folds. This partition process ensured that image ratios (with vs. without pneumothorax) were similar among all training folds. (**B**) After dataset partitioning, images were preprocessed for subsequent training in classification and localization models. (**C**) For the localization model, annotated bounding boxes were first transformed into segmentation masks. The architecture of both classification and localization models was UNet [57] with a backbone of RegNetY [21]. In performing classification tasks, UNet could also output the probability regarding the presence of pneumothorax. Both classification and localization models were trained using Adam optimizer at an initial learning rate of 5e^− 5^. Batch sizes were 8 and 4 for classification and localization models, respectively. (**D** & **E**) Cross entropy loss (**D**) and Dice loss (**E**) were employed for classification model training while Dice loss (**E**) for localization model. UNet [57] and RegNetY [21] were selected based on our pilot experiments. As shown in Supplemental Table 2, we first fixed the segmentation method as UNet [57], varying the backbones and assessing the performance of localization output with the Dice coefficient. RegNetY [21] was selected as the backbone of the segmentation-based system because of the highest Dice coefficient. Subsequently, we set RegNetY [21] as the backbone and evaluated the performance of different segmentation methods. As shown in Supplemental Table 2, UNet [57] was selected due to its compact size and comparable performance to the other methods without significantly increasing the model size

Supplemental Table 1: Results of the pilot experiments in selecting the optimal detectors for the detection-based system

Supplemental Table 2: Results of the pilot experiments in selecting the optimal backbone and segmentation method for the segmentation-based system

Supplemental Table 3: Comparison of images annotated as presence or absence of pneumothorax in training (NTUH-1519) dataset

Supplemental Table 4: Comparison of images annotated as presence or absence of pneumothorax in testing (NTUH-20) dataset

Supplemental Table 5: Computational resources required by computer-aided diagnosis (CAD) systems
