# Supplementary figures and images for: Deep Learning-based Diagnosis and Localization of Pneumothorax on Portable Supine Chest X-ray in Intensive and Emergency Medicine: A Retrospective Study
Source: J Med Syst. 2023 Dec 4;48(1):1. doi: 10.1007/s10916-023-02023-1 (PMC10695857; doi:10.1007/s10916-023-02023-1)

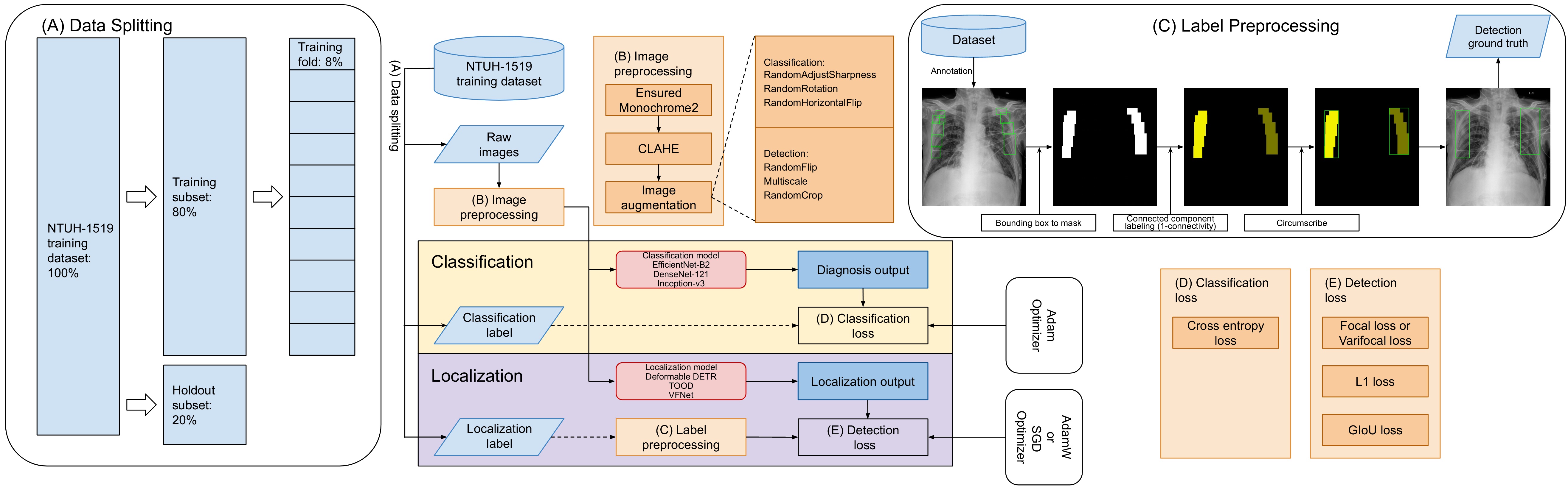

Supplement: Supplementary file 1 — Supplemental Figure 1: Training pipeline for detection-based CAD system: (A) During system development, the training dataset (NTUH-1519) was first randomly split into training (80% of training dataset) and holdout (20% of training dataset) subsets. The training subset was then randomly divided into ten equal-sized validation folds (each 8% of training dataset). This partition process ensured that image ratios (with vs. without pneumothorax) were similar among all validation folds and in the holdout subset. The ten validation folds and the holdout subset were used to identify optimal hyperparameters. (B) Image preprocessing followed dataset partitioning, modifying image intensity to the photometric interpretation of Monochrome2. CLAHE [55] was applied to increase image contrast. Preprocessed images were subsequently passed to classification and localization models for respective training. (C) For the localization model, annotated bounding boxes required further preprocessing, directly transforming the bounding boxes into segmentation masks. The masks were then replaced by one or several larger bounding boxes, covering all adjoining masks within minimum areas for use in training. For classification model, the EfficientNet-B2 [16], DneseNet-121 [15], and Inception-v3 [16] were selected as the model architecture; for localization model, Deformable DETR [17] (backbone: ResNet-50), TOOD [18] (backbone: ResNet-101), and VFNet [19](backbone: ResNet-50) were adopted as the model architecture. The selection of the localization model was based on the comparisons between the state-of-the-art detectors, which were pre-trained on the COCO dataset and fine-tuned on NTUH-1519. We employed commonly used COCO metrics, modifying them to suit the context of image resolution for the assessment. Ultimately, we selected two detectors with the best performance in detecting pneumothorax overall (TOOD and VFNet) and one detector with the best performance in detecting small-sized pneumothorax [file 10916_2023_2023_MOESM1_ESM.jpg]

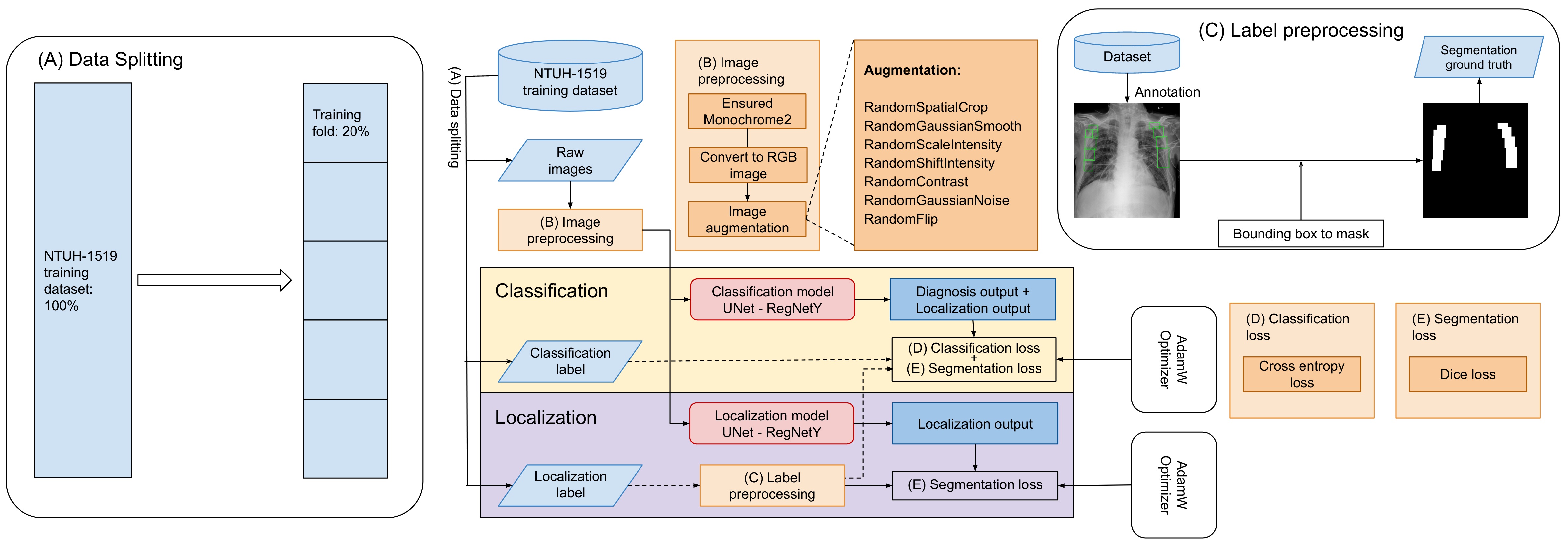

Supplement: Supplementary file 2 — Supplemental Figure 2: Training pipeline for segmentation-based CAD system: (A) During system development, the training dataset (NTUH-1519) was first randomly split into five equal-sized training folds. This partition process ensured that image ratios (with vs. without pneumothorax) were similar among all training folds. (B) After dataset partitioning, images were preprocessed for subsequent training in classification and localization models. (C) For the localization model, annotated bounding boxes were first transformed into segmentation masks. The architecture of both classification and localization models was UNet [57] with a backbone of RegNetY [21]. In performing classification tasks, UNet could also output the probability regarding the presence of pneumothorax. Both classification and localization models were trained using Adam optimizer at an initial learning rate of 5e− 5. Batch sizes were 8 and 4 for classification and localization models, respectively. (D & E) Cross entropy loss (D) and Dice loss (E) were employed for classification model training while Dice loss (E) for localization model. UNet [57] and RegNetY [21] were selected based on our pilot experiments. As shown in Supplemental Table 2, we first fixed the segmentation method as UNet [57], varying the backbones and assessing the performance of localization output with the Dice coefficient. RegNetY [21] was selected as the backbone of the segmentation-based system because of the highest Dice coefficient. Subsequently, we set RegNetY [21] as the backbone and evaluated the performance of different segmentation methods. As shown in Supplemental Table 2, UNet [57] was selected due to its compact size and comparable performance to the other methods without significantly increasing the model size [file 10916_2023_2023_MOESM2_ESM.jpg]
