# Supplementary material for: Deep Learning-based Diagnosis and Localization of Pneumothorax on Portable Supine Chest X-ray in Intensive and Emergency Medicine: A Retrospective Study
Source: J Med Syst. 2023 Dec 4;48(1):1. doi: 10.1007/s10916-023-02023-1 (PMC10695857; doi:10.1007/s10916-023-02023-1)
Supplement: Supplementary file 3 — Supplemental Table 1: Results of the pilot experiments in selecting the optimal detectors for the detection-based system [file 10916_2023_2023_MOESM3_ESM.docx]

**Supplemental Table 1. Results of the pilot experiments in selecting the optimal detectors for the detection-based system**

| **Detector** | **AP** | **AP.5** | **AP.75** | **AP for small pneumothorax** | **AP for medium pneumothorax** | **AP for large pneumothorax** |
| --- | --- | --- | --- | --- | --- | --- |
| VFNet | 0.29 | 0.54 | 0.28 | 0.06 | 0.20 | 0.59 |
| Deformable DETR | 0.22 | 0.44 | 0.22 | 0.08 | 0.13 | 0.51 |
| TOOD | 0.28 | 0.50 | 0.26 | 0.02 | 0.19 | 0.57 |
| Faster R-CNN | 0.22 | 0.42 | 0.2 | 0.01 | 0.09 | 0.53 |
| DETR | 0.23 | 0.40 | 0.22 | 0.01 | 0.13 | 0.52 |
| Pix2Seq | 0.23 | 0.43 | 0.21 | 0.05 | 0.17 | 0.47 |
| YOLOv6s | 0.27 | 0.49 | 0.26 | 0.01 | 0.18 | 0.58 |

AP, average precision; AP.5, average precision at IOU=0.50; AP.75, average precision at IOU=0.75; IOU, intersection over union.
