# Supplementary material for: Deep Learning-based Diagnosis and Localization of Pneumothorax on Portable Supine Chest X-ray in Intensive and Emergency Medicine: A Retrospective Study
Source: J Med Syst. 2023 Dec 4;48(1):1. doi: 10.1007/s10916-023-02023-1 (PMC10695857; doi:10.1007/s10916-023-02023-1)
Supplement: Supplementary file 4 — Supplemental Table 2: Results of the pilot experiments in selecting the optimal backbone and segmentation method for the segmentation-based system [file 10916_2023_2023_MOESM4_ESM.docx]

**Supplemental Table 2. Results of the pilot experiments in selecting the optimal backbone and segmentation method for the segmentation-based system**

| **Backbone** | **Segmentation method** | **Dice** | **Number of parameters (M)** |
| --- | --- | --- | --- |
| *Selection of the backbone* |  |  |  |
| EfficientNetV2s | UNet | 0.580 | 24 |
| DenseNet161 | UNet | 0.574 | 38 |
| ResNet50 | UNet | 0.555 | 32 |
| ResNeXt50 | UNet | 0.557 | 31 |
| SEResNet50 | UNet | 0.573 | 35 |
| ResNeSt50 | UNet | 0.590 | 34 |
| RegNetY | UNet | 0.593 | 25 |
| *Selection of the segmentation method* |  |  |  |
| RegNetY | UNet | 0.593 | 25 |
| RegNetY | DeepLabV3+ | 0.588 | 21 |
| RegNetY | UNet++ | 0.588 | 28 |
| RegNetY | MAnet | 0.596 | 56 |
