# Supplementary material for: Deep Learning-based Diagnosis and Localization of Pneumothorax on Portable Supine Chest X-ray in Intensive and Emergency Medicine: A Retrospective Study
Source: J Med Syst. 2023 Dec 4;48(1):1. doi: 10.1007/s10916-023-02023-1 (PMC10695857; doi:10.1007/s10916-023-02023-1)
Supplement: Supplementary file 5 — Supplemental Table 3: Comparison of images annotated as presence or absence of pneumothorax in training (NTUH-1519) dataset [file 10916_2023_2023_MOESM5_ESM.docx]

**Supplemental Table 3. Comparison of images annotated as presence or absence of pneumothorax in training (NTUH-1519) dataset**

| **Variables** | **Images annotated as pneumothorax (n=490)** | **Images annotated as no pneumothorax (n=1081)** | ***p* -value** |
| --- | --- | --- | --- |
| Age, year | 65.1 (15.6) | 65.1 (16.4) | 0.94 |
| Male, n | 312 (63.7) | 634 (58.6) | 0.06 |
| Qualitative findings in radiology reports, n |  |  |  |
| Atelectasis | 29 (5.9) | 9 (0.8) | <0.001 |
| Cardiomegaly | 150 (30.6) | 506 (46.8) | <0.001 |
| Consolidation | 112 (22.9) | 219 (20.3) | 0.24 |
| Emphysema | 5 (1.0) | 1 (0.09) | 0.006 |
| Endotracheal intubation | 215 (43.9) | 343 (31.7) | <0.001 |
| Haziness | 49 (10.0) | 143 (13.2) | 0.07 |
| Infiltration | 74 (15.1) | 181 (16.7) | 0.41 |
| Nodularity | 23 (4.7) | 32 (3.0) | 0.08 |
| Opacification | 209 (42.7) | 378 (35.0) | 0.003 |
| Pleural effusion | 112 (22.9) | 306 (28.3) | 0.02 |
| Pneumothorax | 484 (98.8) | 0 (0) | <0.001 |

Data expressed as mean (standard deviation) values or as counts (proportions)
