# Supplementary material for: Deep Learning-based Diagnosis and Localization of Pneumothorax on Portable Supine Chest X-ray in Intensive and Emergency Medicine: A Retrospective Study
Source: J Med Syst. 2023 Dec 4;48(1):1. doi: 10.1007/s10916-023-02023-1 (PMC10695857; doi:10.1007/s10916-023-02023-1)
Supplement: Supplementary file 6 — Supplemental Table 4: Comparison of images annotated as presence or absence of pneumothorax in testing (NTUH-20) dataset [file 10916_2023_2023_MOESM6_ESM.docx]

**Supplemental Table 4. Comparison of images annotated as presence or absence of pneumothorax in testing (NTUH-20) dataset**

| **Variables** | **Images annotated as pneumothorax (n=126)** | **Images annotated as no pneumothorax (n=945)** | ***p*-value** |
| --- | --- | --- | --- |
| Age, year | 65.3 (17.8) | 57.7 (20.0) | <0.001 |
| Male, n | 89 (70.6) | 491 (52.0) | <0.001 |
| Qualitative findings in radiology reports, n |  |  |  |
| Atelectasis | 5 (4.0) | 21 (2.2) | 0.23 |
| Cardiomegaly | 31 (24.6) | 255 (27.0) | 0.57 |
| Consolidation | 18 (14.3) | 59 (6.2) | 0.001 |
| Emphysema | 3 (2.4) | 1 (0.1) | <0.001 |
| Endotracheal intubation | 45 (35.7) | 90 (9.5) | <0.001 |
| Haziness | 6 (4.8) | 66 (7.0) | 0.35 |
| Infiltration | 35 (27.8) | 169 (17.9) | 0.008 |
| Nodularity | 5 (4.0) | 41 (4.3) | 0.85 |
| Opacification | 78 (61.9) | 301 (31.9) | <0.001 |
| Pleural effusion | 34 (27.0) | 166 (17.6) | 0.01 |
| Pneumothorax | 115 (91.3) | 9 (1.0) | <0.001 |

Data expressed as mean (standard deviation) values or as counts (proportions)
