# Supplementary material for: Deep Learning-based Diagnosis and Localization of Pneumothorax on Portable Supine Chest X-ray in Intensive and Emergency Medicine: A Retrospective Study
Source: J Med Syst. 2023 Dec 4;48(1):1. doi: 10.1007/s10916-023-02023-1 (PMC10695857; doi:10.1007/s10916-023-02023-1)
Supplement: Supplementary file 7 — Supplemental Table 5: Computational resources required by computer-aided diagnosis (CAD) systems [file 10916_2023_2023_MOESM7_ESM.docx]

**Supplemental Table 5. Computational resources required by computer-aided diagnosis (CAD) systems**

| **Model** | **Type** | **Number of parameters (M)** | **Number of flops (G)** | **Inference GPU memory usage (GB)** | **Computational resources** |
| --- | --- | --- | --- | --- | --- |
| *Detection-based CAD system* | | | | | |
| Inception-v3 | Classification | 21.79 | 74.87 | 0.87 | The detection-based CAD system was trained on an Ubuntu 18.04.6 LTS operating system, loaded with PyTorch 1.10.0 deep learning framework ^53^ and MMDetection 2.21.0 object detection toolbox ^54^. Hardware required for training included eight 2.20-GHz Intel(R) Xeon(R) Gold 5220 CPUs, 128 GB hard disk space, 503 GB RAM, and one NVIDIA Quadro RTX 8000 PCIe graphics processing unit equipped with CUDA 11.3 (Nvidia Corp, Santa Clara, CA). |
| DenseNet-121 | Classification | 6.95 | 60.22 | 3.00 |  |
| EfficientNet-B2 | Classification | 7.70 | 14.12 | 2.26 |  |
| Deformable DETR (R-50) | Detection | 39.82 | 195.23 | 2.21 |  |
| TOOD (R-101) | Detection | 50.79 | 256.58 | 2.17 |  |
| VFNet (R-50) | Detection | 32.48 | 188.97 | 2.19 |  |
| *Segmentation-based CAD system* | | | | | |
| UNet-RegNetY | Classification | 25 | - | 2.10 | The segmentation-based model was trained on an Ubuntu 20.04.4 LTS operating system, loaded with PyTorch 1.10.2 deep learning framework ^53^ and with CUDA 11.6. Training required four 2.20-GHz Intel(R) Xeon(R) E5-2650 v4 CPUs, 128 GB hard disk space, 16 GB RAM, and one Tesla P100-PCIE-16GB graphics processing unit (Nvidia Corp). |
| UNet-RegNetY | Segmentation | 25 | - | 2.10 |  |
